# Comparison of nomogram and machine‐learning methods for predicting the survival of non‐small cell lung cancer patients

**DOI:** 10.1002/cai2.24

**Published:** 2022-08-30

**Authors:** Haike Lei, Xiaosheng Li, Wuren Ma, Na Hong, Chun Liu, Wei Zhou, Hong Zhou, Mengchun Gong, Ying Wang, Guixue Wang, Yongzhong Wu

**Affiliations:** ^1^ Chongqing Key Laboratory of Translational Research for Cancer Metastasis and Individualized Treatment Chongqing University Cancer Hospital Chongqing China; ^2^ Digital Health China Technologies, Co., Ltd. Beijing China; ^3^ MOE Key Lab for Biorheological Science and Technology, State and Local Joint Engineering Laboratory for Vascular Implants College of Bioengineering Chongqing University Chongqing China

**Keywords:** nomogram, machine learning, non‐small cell lung cancer, overall survival, predictive model

## Abstract

**Background:**

Most patients with advanced non‐small cell lung cancer (NSCLC) have a poor prognosis. Predicting overall survival using clinical data would benefit cancer patients by allowing providers to design an optimum treatment plan. We compared the performance of nomograms with machine‐learning models at predicting the overall survival of NSCLC patients. This comparison benefits the development and selection of models during the clinical decision‐making process for NSCLC patients.

**Methods:**

Multiple machine‐learning models were used in a retrospective cohort of 6586 patients. First, we modeled and validated a nomogram to predict the overall survival of NSCLC patients. Subsequently, five machine‐learning models (logistic regression, random forest, XGBoost, decision tree, and light gradient boosting machine) were used to predict survival status. Next, we evaluated the performance of the models. Finally, the machine‐learning model with the highest accuracy was chosen for comparison with the nomogram at predicting survival status by observing a novel performance measure: time‐dependent prediction accuracy.

**Results:**

Among the five machine‐learning models, the accuracy of random forest model outperformed the others. Compared with the nomogram for time‐dependent prediction accuracy with a follow‐up time ranging from 12 to 60 months, the prediction accuracies of both the nomogram and machine‐learning models changed as time varied. The nomogram reached a maximum prediction accuracy of 0.85 in the 60th month, and the random forest algorithm reached a maximum prediction accuracy of 0.74 in the 13th month.

**Conclusions:**

Overall, the nomogram provided more reliable prognostic assessments of NSCLC patients than machine‐learning models over our observation period. Although machine‐learning methods have been widely adopted for predicting clinical prognoses in recent studies, the conventional nomogram was competitive. In real clinical applications, a comprehensive model that combines these two methods may demonstrate superior capabilities.

## Graphical abstract

Thirteen clinical variables related to survival status were selected for modeling and analysis. During our observation period, nomograms provided a more reliable prognostic assessment of NSCLC patients compared to machine‐learning models. In practical clinical applications, an integrated model combining these two approaches may demonstrate superior capabilities.

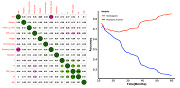

**Abbreviations**
AJCC: American Joint Committee on Cancer
AUC: The area under the curve
CUCH: The Cancer Hospital Affiliated to Chongqing University
DCA: decision curve analysis
KM: Kaplan–Meier
KPS: Karnofsky Performance Scale
NSCLC: non‐small cell lung cancer
OS: overall survival
ROC: receiver operating characteristics
SEER: the surveillance, epidemiology, and end results

## 1 BACKGROUND

Lung cancer is the leading cause of cancer‐related death worldwide. Non‐small cell lung cancer (NSCLC) accounts for 83% of all lung cancer cases, with an incidence rate of 40.60 per 100,000 and a 5‐year survival rate of only 22.1% [1]. Although significant progress has been made in molecular targeted therapy and immunotherapy, which has improved the quality of life and survival rates of patients, their prognoses remain poor [2]. Thus, it is particularly important to determine the prognosis of NSCLC patients to develop optimal treatment regimens. Currently, clinicians usually determine prognosis on the basis of surgical pathological staging, which only considers the primary tumor, regional lymph node involvement, and distant metastasis. This ignores the role of other prognostic factors, making it difficult to obtain satisfactory predictive results [3]. Therefore, there is an urgent need for a clinical prognostic assessment system with highly reliable predictive capability for NSCLC patients.

Nomograms are commonly used tools for estimating prognosis in oncology and other medical fields. For survival data, the underlying model on which the nomogram is based is typically the Cox proportional hazards model, which models the relationship between a set of covariates and the hazard function of a particular failure time [4]. With the ability to generate an individual numerical probability of a clinical event by integrating diverse prognostic and determinant variables, the nomogram fulfills our desire for biologically‐ and clinically integrated models and the goal of personalized medicine [5]. Nomograms have been widely used to estimate cancer prognosis [6] because of their high accuracy, flexibility, and ease of generalization compared with traditional prognostic models such as Cox regression and logistic regression [7]. Nomograms have also been used to analyze clinical data of various tumor types, including liver, bladder, prostate, cervical, and gastric cancers [8, 9, 10, 11, 12]. Owing to their intuitive and easy‐to‐understand features, nomograms have gradually gained increased use in medical research and clinical practice and have been widely used to predict the survival of cancer patients [13, 14, 15].

Machine‐learning algorithms are another method used to estimate prognosis because they can learn quickly from a large amount of patient data to produce more accurate predictions than a set of clinical experts [16]. Machine‐learning models have been increasingly used for cancer prognostics in recent years. The first study to use machine learning of administrative and registry data for cancer survival prediction was published in 2014 [17]. The machine‐learning models and clinicians separately estimated patient survival status by producing a probability for each of three time periods: 6 months, 1 year, and 2 years. Predictive performances were then measured using the area under the curve (AUC) analysis. The machine‐learning model outperformed clinicians, and the AUC of the prediction showed a downward trend as time increased [17]. Parikh et al. [18] found that machine‐learning algorithms (logistic regression, gradient boosting machine [GBM], and random forest) built on structured real‐time electronic health record data performed adequately at identifying outpatients with cancer who had a high risk of short‐term mortality, suggesting that machine‐learning tools hold promise for integration into clinical workflows to ensure that patients with cancer have timely conversations about their goals and values. Ding et al. [19] recently developed a miRNA‐based machine‐learning prediction model for cervical cancer survival that robustly stratified cervical cancer patients into one of three categories: high survival rate (5‐year survival rate ≥ 90%), moderate survival rate (5‐year survival rate ≈ 65%), and low survival rate (5‐year survival rate ≤ 40%). Lee et al. [20] developed a novel machine‐learning‐based approach that produced a prognostic model called Survival Quilts, which could discriminate 10‐year prostate cancer‐specific mortality similar to the top‐ranked prognostic models using only standard clinicopathological variables. Many studies have assessed the survival of lung cancer patients by analyzing large data sets using machine‐learning techniques, including logistic regression and support vector machines [21], as well as methods based on integrated clustering [22]. Artificial neural networks have been used to predict the survival of patients with NSCLC and showed an overall accuracy of 83% [23]. Parsimonious Bayes and decision trees have also been used to predict the survival of lung cancer patients with an accuracy of 90% [24].

The aim of this study was to use a dynamic labeling method to compare the performance of nomograms based on the Cox proportional hazards model with machine‐learning methods at predicting the overall survival (OS) of NSCLC patients. The input data comprised a large follow‐up data set from the Cancer Hospital Affiliated to Chongqing University (CUCH), China. Follow‐up times were calculated from the date a patient initiated treatment to the date of their last follow‐up or death. The dynamic labeling method used in this study provides a novel perspective on the basis of timeline observations to compare the performance of nomograms and machine‐learning methods for prognostic predictions.

## 2 METHODS

### 2.1 Study population

We selected patients who were admitted to the CUCH between 2013 and 2020 in accordance with the following inclusion criteria: (1) diagnosed with NSCLC, (2) complete patient medical records were available, and (3) age ≥ 18 years. Patients who were repeatedly admitted were excluded, and all personal information was deidentified. The clinical and pathologic characteristics included age, sex, weight, smoking history, Karnofsky Performance Scale (KPS) score according to the Karnofsky Performance Scale Index, which is an assessment tool for functional impairment [25], tumor stage according to the American Joint Committee on Cancer (AJCC) Seventh edition [26], and treatment (surgery, radiotherapy, and chemotherapy). The follow‐up time (in months), which was from treatment initiation until the last follow‐up or death [27], and the survival status of the patients were also included.

### 2.2 Statistical analysis

The *χ* ^2^ test was used for categorical variables (Fisher's exact test was used for expected values < 5) and the Student's *t* test or Mann‐Whitney U test as appropriate was used for between‐group comparisons of continuous variables. The median follow‐up time and its 95% confidence interval were estimated by Kaplan–Meier (KM) analysis, and differences between training and validation groups were analyzed by the log‐rank test. All analyses were performed using R (version 4.0.3) and R Studio (version 1.3.1093) software.

### 2.3 Feature selection method

First, pairwise Spearman's rank correlation was performed [28]. For two variables that were highly correlated (absolute value of correlation coefficient > 0.8) [29], only one was used for further modeling and the other was removed. The most relevant features for the outcome from the remaining variables were selected using the Boruta method [30, 31]. Follow‐up time was used as a feature for machine‐learning modeling [32]. Accordingly, features selected in accordance with the above steps were used in both the machine‐learning models and nomograms.

### 2.4 Development and validation of the prognostic nomogram

Multivariate Cox regression analysis was applied to variables obtained using the Boruta method to determine whether they were independent factors. A Cox regression model‐based nomogram was used to predict the 1‐, 3‐, and 5‐year OS rates of NSCLC patients. R software (version 4.0.3) was used to construct a survival prediction model for NSCLC patients on the basis of the Cox proportional hazards. The concordance index (C‐index) and calibration curve were used to evaluate the accuracy and predictive ability of the nomogram, respectively. The C‐index ranges from 0.5 to 1, where 1 indicates complete discrimination and 0.5 means no discrimination. This metric was used to measure the performance of the nomogram. A value greater than 0.7 usually indicates a relatively good distinction, and the closer the C‐index is to 1, the better the predictive accuracy. A calibration curve was used to compare the consistency between the predicted results of the nomogram and the actual results. Identical results would lead to the calibration curve coinciding with the diagonal. The self‐sampling and replacement method (bootstrapping) was applied to internally verify the model and avoid overfitting. The fitted line of the calibration curve would only coincide with the reference line if the predicted value was equal to the actual value. If the predicted value was greater than the actual value, that is, the risk was overestimated, the fitted line would be below the reference line.

Receiver operating characteristics (ROC), which considers specificity and sensitivity, and decision curve analysis, which can calculate net benefits [33], were used to evaluate the prediction results.

### 2.5 Development and evaluation of machine‐learning models

We used survival status (dead/alive) of the last follow‐up time as the predictor class and incorporated the following methods for the classification model: logistic regression, random forest, XGBoost, decision tree, and Light Gradient Boosting Machine (lightGBM). The extracted data were checked to ensure that these were properly preprocessed, and all the variables were converted to numeric to reduce possible spelling errors. For model development and evaluation, we randomly assigned 70% and 30% of patients into the training and validation groups, respectively. All analyses were performed using R software (version 4.0.3).

The training and hyperparameter tuning of machine learning models were implemented on 70% of the data (training set) using a ten‐fold cross‐validation method. The remaining 30% of the data were used to compare the performance of machine learning models. After training, the algorithms were evaluated for the performance metrics of interest. As our machine‐learning models were binary classifiers, they were evaluated using the accuracy, AUC, precision, recall and F1‐score. The four possible outcomes, true positive (TP), true negative (TN), false positive (FP), and false negative (FN), were the basis of these evaluation measures. Accuracy was the fraction of correct predictions, both true positives and true negatives among all subjects. AUC was computed by plotting sensitivity against 1 − specificity for all possible cutoff points and was calculated as an overall measure of the discrimination abilities of the models. Higher AUC values indicated better model performance. Recall represents the proportion of patients who were correctly identified as nonsurvivors by our classifier among all nonsurviving patients (also known as the “true positive” rate). Specificity refers to the proportion of patients who were correctly predicted to survive among those who actually survived (also known as the “true negative” rate). A higher precision means that the classifier resulted in more TP than FP results. F1‐score is the harmonic mean of a system's precision and recall values.

(1) $$\mathrm{Accuracy}\;=\;\frac{\mathrm{TP}\;+\;\mathrm{TN}}{\mathrm{TP}\;+\;\mathrm{TN}\;+\;\mathrm{FP}\;+\;\mathrm{FN}},$$

(2) $$\mathrm{Precision}\;=\;\frac{\mathrm{TP}}{\mathrm{TP}\;+\;\mathrm{FP}},$$

(3) $$\mathrm{Recall}\;=\;\frac{\text{TP}}{\mathrm{TP}\;+\;\mathrm{FN}},$$

(4) $$\text{F1‐Score}\;=\;\frac{2\times \text{Precision}\times \;\text{Recall}}{\mathrm{TP}\;+\;\mathrm{FP}}$$

### 2.6 Comparing the nomogram and machine‐learning methods

To better compare the results of the various methods, we re‐labeled the survival status of each patient in the validation cohort at each timepoint. For example, at the 5‐year follow‐up timepoint, a patient who was labeled as dead had died within 5 years from the first treatment. Similarly, individuals who were labeled as alive were alive for at least 5 years after the first treatment. Patients who were lost to follow‐up before a certain timepoint were excluded from the validation cohort. At each timepoint, a new relabeled validation set was generated to evaluate the accuracy of the model according to Equation (1).

Using dynamic labeling, we generated a time‐dependent accuracy curve to compare the predictive performances of the nomogram and machine‐learning models. The machine‐learning model with the highest accuracy (according to Equation 1) among the five models (logistic regression, random forest, XGBoost, decision tree, and lightGBM) was selected for comparison with the nomogram.

## 3 RESULTS

### 3.1 Patient characterization

The study cohort included 6586 NSCLC patients, 4337 males and 2249 females, with a male‐to‐female ratio of 1.93:1. The mean age at diagnosis was 61.6 years (standard deviation: ±10.3; range: 23–99 years), and the median age was 62.0 years. The NSCLC patients were randomly assigned to the training group (70%) and validation group (30%). The demographic and clinicopathological characteristics of the patients are shown in Table 1.

**Table 1:** Patient characteristics in the study (*n* = 6586)

Variable	Training set (*n* = 4610)	Validation set (*n* = 1976)	*p* value
Age (years)^a^	61.4 ± 10.3	62.0 ± 10.3	0.01*
Sex, n(%)			0.58
Male	3046 (66.07)	1291 (65.33)	
Female	1564 (33.93)	685 (34.67)	
Weight (kg)^a^	58.8 ± 10.1	58.8 ± 10.0	0.78
Follow‐up time (months)^a^	36.7 (31.6−37.7)	36.1 (28.6−37.9)	0.69
Smoking history, n(%)			0.27
No	2153 (46.70)	953 (48.23)	
Yes	2457 (53.30)	1023 (51.77)	
KPS score	78.8 ± 10.4	78.6 ± 10.3	0.27
T, n(%)			0.59
Tis	57 (1.24)	22 (1.11)	
T1	737 (15.99)	336 (17.00)	
T2	1190 (25.81)	517 (26.16)	
T3	718 (15.57)	301 (15.23)	
T4	1736 (37.66)	713 (36.08)	
TX	172 (3.73)	87 (4.40)	
N, n(%)			0.07
N0	1175 (25.49)	551 (27.88)	
N1	394 (8.55)	153 (7.74)	
N2	972 (21.08)	406 (20.55)	
N3	1855 (40.24)	754 (38.16)	
NX	214 (4.64)	112 (5.67)	
M, n(%)			0.35
M0	1678 (36.40)	746 (37.75)	
M1	2847 (61.76)	1187 (60.07)	
MX	85 (1.84)	43 (2.18)	
Surgery, n(%)			1.00
No	881 (19.11)	377 (19.08)	
Yes	3729 (80.89)	1599 (80.92)	
Radiation, n(%)			0.77
No	3384 (73.41)	1443 (73.03)	
Yes	1226 (26.59)	533 (26.97)	
Chemotherapy, n(%)			0.63
No	2360 (51.19)	998 (50.51)	
Yes	2250 (48.81)	978 (49.49)	

Abbreviations: KPS, Karnofsky Performance Scale; M, refers to whether cancer has metastasized; N, refers to the number of nearby lymph nodes that are cancerous; T, refers to the size and extent of the main tumor.

^a^ Age and weight were expressed as mean ± standard deviation, and follow‐up time was expressed as median (95% confidence interval). *p < 0.05 indicating statistical significance.

### 3.2 Selected features

Pairwise Spearman's rank correlations were calculated for the 13 variables that were related to survival status (Supporting Information: Figure S1). M stage and TNM stage were highly correlated (correlation coefficient = 0.84); thus, only M stage was used for further model development and TNM stage was removed. The 12 remaining features were selected by the Boruta method, and all were confirmed to be important (Supporting Information: Table S1). Follow‐up time was the most important feature, while radiation therapy ranked last (Supporting Information: Figure S2). The order of feature importance was as follows: follow‐up time, N stage, KPS score, M stage, surgery, T stage, chemotherapy, smoking history, age, sex, weight, and radiation therapy. These 12 features were used to construct the nomogram and machine‐learning models. As the nomogram was constructed based on Cox regression which investigates the association between features and survival time of patients to predict hazard ratio while handling the censoring of observations, follow‐up time was not used as a feature in constructing the nomogram.

### 3.3 Multivariate Cox regression analysis results

**Figure 1:**
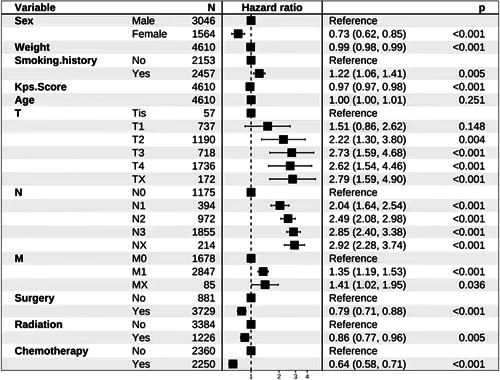
Multivariate Cox regression analysis of overall survival in non‐small cell lung cancer patients

Multivariate Cox regression analysis showed that all variables included in the regression were significantly related to outcome and that all were independent factors affecting the prognosis of NSCLC patients (*p* < 0.001) (Figure 1). Patient prognosis worsened as the degree of T, N, and M increased, while patients who had undergone surgery, radiotherapy, or chemotherapy had better prognoses.

### 3.4 Nomogram analysis and validation results

**Figure 2:**
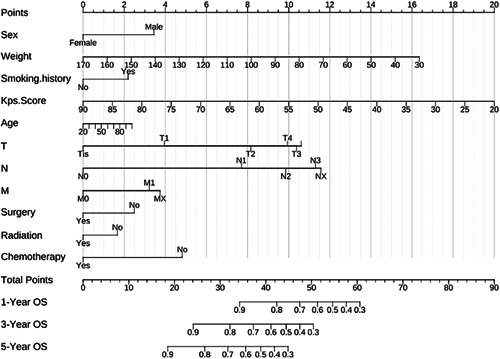
Nomogram for predicting 1‐, 3‐ and 5‐year overall survival

A nomogram incorporating all significant independent factors for predicting 1‐, 3‐, and 5‐year OS rates in the training cohort was established on the basis of the selected variables in accordance with their hazard ratios. Each nomogram was used by first scoring each variable on its corresponding point scale. The scores of all variables were then added to obtain the total points, and a vertical line was dropped from the row of total points to estimate 1‐, 3‐, and 5‐year survival rates (Figure 2).

The C‐index of the nomogram was 0.74 (95% confidence interval: 0.73–0.75). Calibration curves for patients who underwent NSCLC surgery were plotted in Supporting Information: Figure S3, with the blue, red, and green lines fitted for 1‐, 3‐, and 5‐year OS, respectively. As indicated, the calibration curves are relatively close to the diagonal.

**Figure 3:**
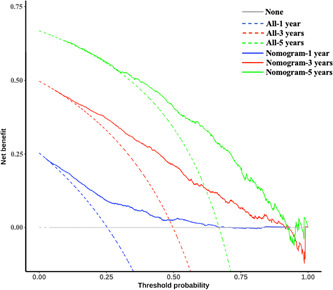
Decision curve analysis of the nomogram in the validation cohort for 1‐, 3‐ and 5‐year overall survival

The nomogram yielded net benefits (Figure 3), as 1‐, 3‐, and 5‐year decision curve analysis of the nomogram indicated net benefits for OS compared with the treat‐all strategy (blue, red, and green dashed lines) or treat‐none strategy (gray line).

### 3.5 Evaluation of results from the machine‐learning models

The predictive accuracy and model performance evaluation of the five machine‐learning algorithm models are presented in Table 2 and Supporting Information: Figure S4. The random forest algorithm showed the highest accuracy of 0.702, the highest recall of 0.813, and the highest F1‐score of 0.763.

**Table 2:** Performance metrics of the cross‐validated machine‐learning method in the validation cohort

Model	Accuracy	AUC	Precision	Recall	F1‐score
Logistic regression	0.684	0.757	0.712	0.779	0.744
Random forest	0.702	0.770	0.719	0.813	0.763
XGBoost	0.691	0.770	0.719	0.782	0.749
Decision tree	0.698	0.731	0.719	0.801	0.758
LightGBM	0.697	0.773	0.764	0.707	0.735

Abbreviations: AUC, area under the curve; LightGBM, light gradient boosting machine.

### 3.6 Comparison of results from the nomogram and random forest algorithm

**Figure 4:**
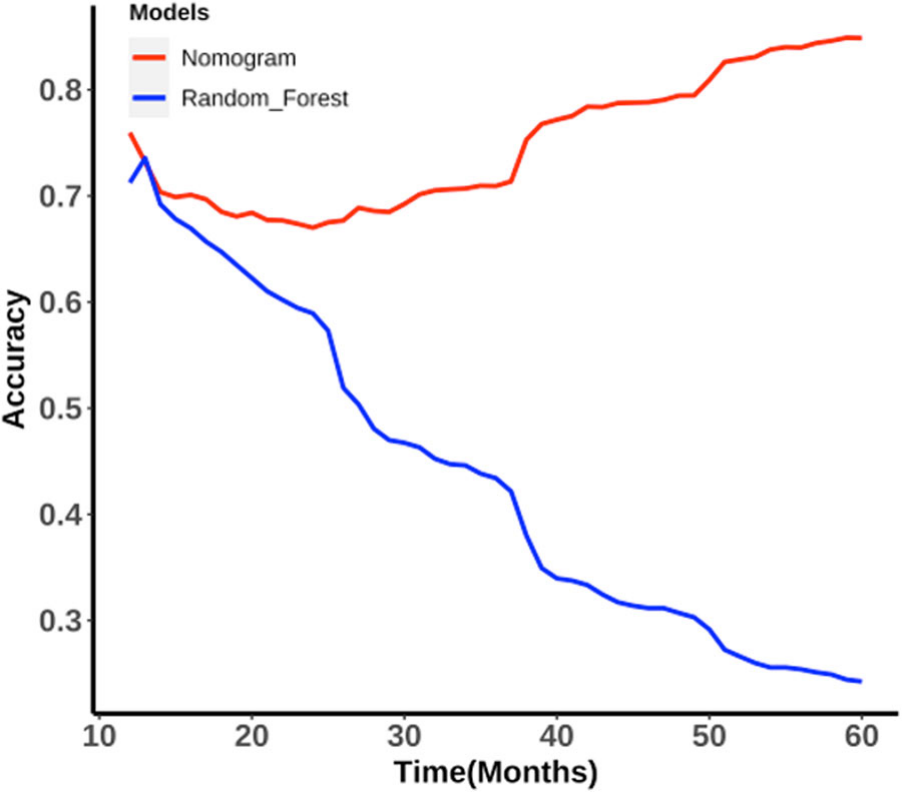
Time‐dependent accuracy of the nomogram and random forest algorithm

For follow‐up times ranging from the 12th month (1 year) to the 60th month (5 years), we relabeled the survival status of the validation cohort and evaluated predictive accuracy (according to Equation 1) for each month, and then determined the time‐dependent accuracy (Figure 4 and Supporting Information: Table S2). The accuracy of nomogram predictions showed a downward trend between the 12th and 24th months and an increasing trend between the 24th and 60th months (Figure 4). At the 60th month, the nomogram reached its maximum predictive accuracy, which was 0.85 (Figure 4). The random forest algorithm with optimum parameters (mtry = 4, maxnodes = 31, ntree = 2000) reached its maximum predictive accuracy of 0.74 at the 13th month and decreased thereafter. From the 12th to the 60th months, the predictive accuracy of the nomogram was superior to than that of the random forest algorithm except the 13th month (Figure 4).

## 4 DISCUSSION

In this study, we first selected variables related to OS using the Boruta method. All 12 independent variables were confirmed to be important and were further used to construct the nomogram and machine‐learning models. We then used the Cox regression model to analyze and build a nomogram that predicted 1‐, 3‐, and 5‐year OS rates of the patients. The C‐index and calibration curves of the nomogram showed good predictive ability. We then compared five machine‐learning models for predicting patient survival status, of which the random forest model achieved the best performance. The model with the highest accuracy was compared with the nomogram using the dynamic labeling validation cohort and time‐dependent accuracy (Supporting Information: Table S2). To ensure that the best model was determined dynamically at different timepoints, our study proposed a novel comparison method that adopted dynamic labeling and time‐dependent accuracy to compare the performances of the nomogram and machine‐learning models.

The C‐index of the nomogram for OS in our study was 0.74 (95% confidence interval: 0.73–0.75), which showed that the model could accurately predict survival status. For the calibration curve, the horizontal coordinates of the graph represent the probability of occurrence predicted by our model; the vertical coordinates represent the actual incidence in the patients. In this study, all three fitted lines corresponded well with the reference line, indicating that the prediction model provided a high accuracy.

Logistic regression, random forest, XGBoost, decision tree, and lightGBM were selected to build the machine‐learning prognostic models. Random forest achieved the best performance in terms of accuracy, recall and F1‐score. Furthermore, regarding the use of the model in a clinical setting, patients with FP results (i.e., the survivor is predicted as nonsurvivor) may be overtreated, while those with FN results (i.e., nonsurvivors are predicted to be survivors) may miss timely actions for early prevention/treatment. Thus, accuracy is extremely important when developing prediction models for clinical use [34, 35].

To date, only one study has compared the prognostic accuracy of nomograms with machine‐learning models. Alabi et al. [32] compared the performance of a nomogram with a machine‐learning model to predict OS in patients with tongue cancer using the Surveillance, Epidemiology, and End Results (SEER) program database. However, survival status was only predicted at one follow‐up timepoint (5 years) in that study. As survival status is time‐dependent, we believe that time‐dependent accuracy should be considered when comparing models. Thus, we compared the accuracy of the nomogram and machine‐learning models at 48 timepoints (Supporting Information: Table S2), from the 12th to the 60th month, using the dynamic labeling method and the dynamic validation cohort. Together, this provided stronger evidence for the advancement of the nomogram in predicting survival status.

This study has several limitations. First, for some variables used in this study (such as KPS score), we cannot find corresponding variables in outside public databases (including SEER) to externally validate the nomogram and predictions. The predictive performances of the Cox regression model and the best‐performing machine‐learning model could also be improved in some ways. For example, as the number of patients or variables increases, the results of both methods may improve. Additionally, improved model adaptability is a future aim. Combining more than one model by integrating rule‐based knowledge to predict and generate a comprehensive result may be a potential solution to achieve the best performance in clinical decision‐making.

## 5 CONCLUSIONS

In this study, we compared a nomogram with machine‐learning methods to predict the survival of NSCLC patients. The nomogram outperformed the random forest model (which performed the best among the five machine‐learning methods) at 47 timepoints except the 13th month. Although machine‐learning methods have recently been widely adopted, nomograms remain competitive prognostic predictive tools. Therefore, a solution to prognostic analysis may include combining the nomogram and machine‐learning methods to provide objective and comprehensive clinical decision‐making for the personalized treatment of NSCLC patients.

## AUTHOR CONTRIBUTIONS

Haike Lei and Xiaosheng Li contributed equally to the study. Guixue Wang and Yongzhong Wu were responsible for study design and conception; Haike Lei and Xiaosheng Li collected or contributed to the data; Wuren Ma, Na Hong, Chun Liu, and Wei Zhou were responsible for data processing and data analysis; Hong Zhou, Mengchun Gong, and Ying Wang interpreted the results; all authors drafted the manuscript. All authors revised the manuscript for important intellectual content.

## CONFLICT OF INTEREST

All authors declare that there is no conflict of interest except Professor Mengchun Gong, who is a member of the *Cancer Innovation* Editorial Board. To minimize bias, he was excluded from all editorial decision‐making related to the acceptance of this article for publication.

## ETHICS STATEMENT

The authors are accountable for all aspects of the work in ensuring that questions related to the accuracy or integrity of any part of the work are appropriately investigated and resolved. The present study was performed according to the guidelines of the Declaration of Helsinki and was approved by the Ethics Committee of the Chongqing University Cancer Hospital (CZLS2022061‐A). All data identifying the patient's personal information were deleted.

## INFORMED CONSENT

The need for informed consent was waived by the Affiliated Chongqing University Cancer Hospital Ethics Committee, given the retrospective nature of the study.

## Supporting information

Supporting information.

## Data Availability

The data used in this manuscript are for research purposes only and are not publicly available because they are in‐hospital patient data.
