# Supplementary material for: Comparison of nomogram and machine‐learning methods for predicting the survival of non‐small cell lung cancer patients
Source: Cancer Innov. 2022 Aug 30;1(2):135–45. doi: 10.1002/cai2.24 (PMC10686174; doi:10.1002/cai2.24)
Supplement: Supplementary file 1 — Supporting information. [file CAI2-1-135-s001.docx]

**
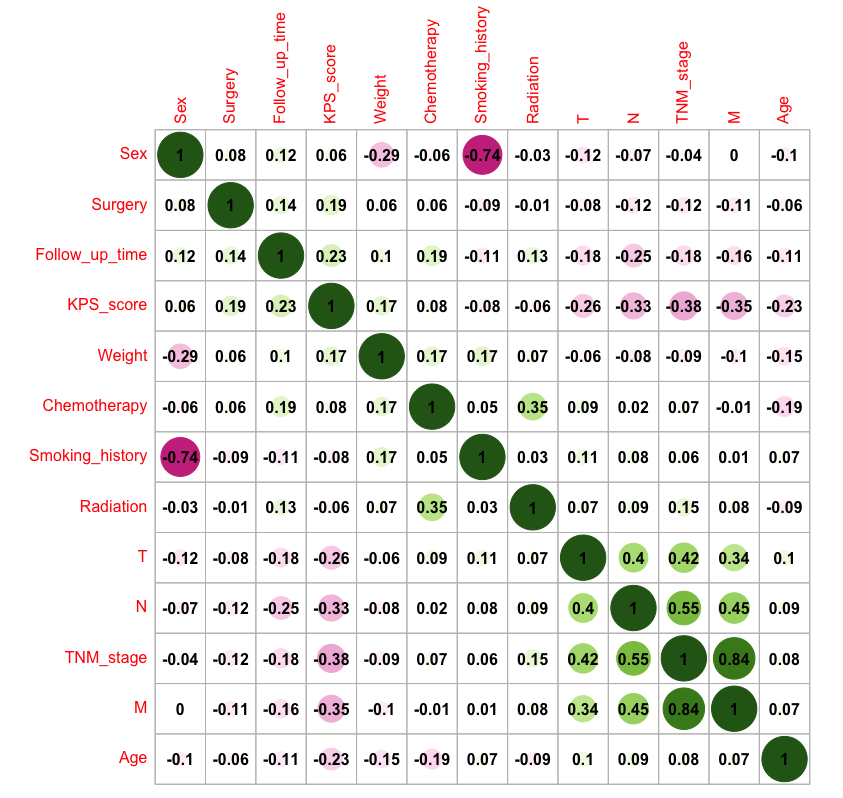
Figure S 1 Correlation matrix of patient characteristics.**

**
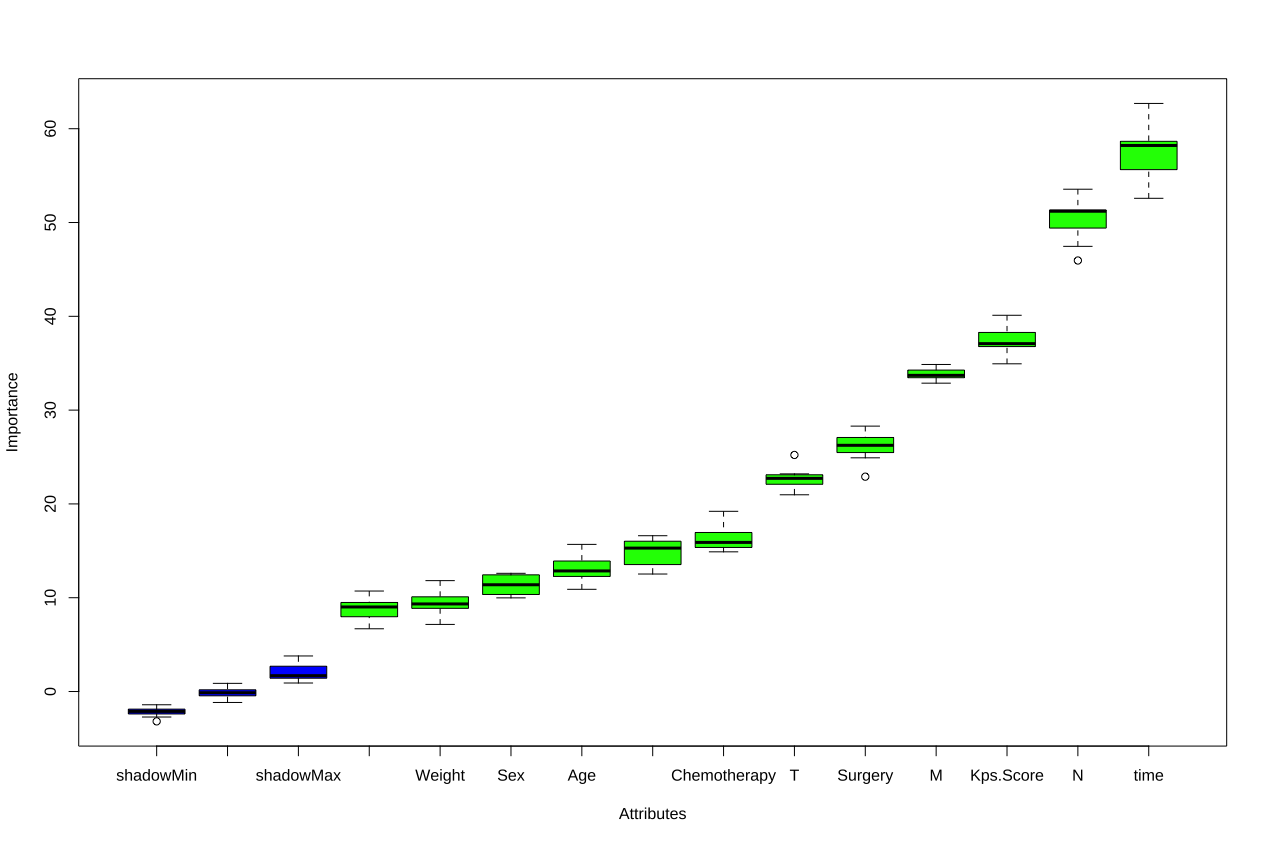
****Figure S 2 Feature importance ranked by boruta method.**

| **Table S 1 Feature importance ranking and selection decision by boruta method.** | | | | | | |
| --- | --- | --- | --- | --- | --- | --- |
| **Features** | **meanImp** | **medianImp** | **minImp** | **maxImp** | **normHits** | **decision** |
| **Follow up time** | 57.50 | 58.21 | 52.58 | 62.69 | 1 | Confirmed |
| **N** | 50.22 | 51.20 | 45.95 | 53.55 | 1 | Confirmed |
| **Kps score** | 37.52 | 37.09 | 34.94 | 40.11 | 1 | Confirmed |
| **M** | 33.81 | 33.71 | 32.87 | 34.87 | 1 | Confirmed |
| **Surgery** | 26.07 | 26.25 | 22.90 | 28.29 | 1 | Confirmed |
| **T** | 22.64 | 22.73 | 20.97 | 25.22 | 1 | Confirmed |
| **Chemotherapy** | 16.37 | 15.90 | 14.89 | 19.21 | 1 | Confirmed |
| **Smoking history** | 14.81 | 15.29 | 12.53 | 16.61 | 1 | Confirmed |
| **Age** | 13.05 | 12.86 | 10.90 | 15.69 | 1 | Confirmed |
| **Sex** | 11.42 | 11.38 | 9.98 | 12.60 | 1 | Confirmed |
| **Weight** | 9.42 | 9.34 | 7.15 | 11.82 | 1 | Confirmed |
| **Radiation therapy** | 8.83 | 9.01 | 6.69 | 10.71 | 1 | Confirmed |

meanImp: mean importance; medianImp: median importance; minImp: min importance; maxImp: max importance

| **Table S 2 Model accuracy and patient number in validation set at different follow up time points.** | | | | | | |
| --- | --- | --- | --- | --- | --- | --- |
| **Follow up time point**  **(month)** | **Nomogram accuracy** | **Random forest accuracy** | **Patient number in validation set** | **Patient number excluded from validation set** | **Patient number labelled dead** | **Patient number labelled alive** |
| 12 | 0.760 | 0.713 | 1680 | 296 | 456 | 1224 |
| 13 | 0.732 | 0.735 | 1600 | 376 | 489 | 1111 |
| 14 | 0.704 | 0.692 | 1488 | 488 | 548 | 940 |
| 15 | 0.699 | 0.678 | 1448 | 528 | 569 | 879 |
| 16 | 0.701 | 0.669 | 1428 | 548 | 580 | 848 |
| 17 | 0.697 | 0.657 | 1405 | 571 | 590 | 815 |
| 18 | 0.685 | 0.647 | 1378 | 598 | 599 | 779 |
| 19 | 0.681 | 0.635 | 1356 | 620 | 608 | 748 |
| 20 | 0.684 | 0.623 | 1333 | 643 | 620 | 713 |
| 21 | 0.677 | 0.609 | 1311 | 665 | 627 | 684 |
| 22 | 0.677 | 0.602 | 1297 | 679 | 633 | 664 |
| 23 | 0.674 | 0.595 | 1275 | 701 | 638 | 637 |
| 24 | 0.670 | 0.589 | 1264 | 712 | 645 | 619 |
| 25 | 0.675 | 0.572 | 1225 | 751 | 652 | 573 |
| 26 | 0.677 | 0.519 | 1148 | 828 | 689 | 459 |
| 27 | 0.689 | 0.504 | 1128 | 848 | 699 | 429 |
| 28 | 0.686 | 0.479 | 1111 | 865 | 711 | 400 |
| 29 | 0.685 | 0.469 | 1098 | 878 | 715 | 383 |
| 30 | 0.692 | 0.467 | 1089 | 887 | 716 | 373 |
| 31 | 0.701 | 0.463 | 1082 | 894 | 717 | 365 |
| 32 | 0.705 | 0.452 | 1072 | 904 | 721 | 351 |
| 33 | 0.706 | 0.448 | 1062 | 914 | 724 | 338 |
| 34 | 0.707 | 0.445 | 1058 | 918 | 725 | 333 |
| 35 | 0.710 | 0.439 | 1047 | 929 | 728 | 319 |
| 36 | 0.709 | 0.434 | 1039 | 937 | 729 | 310 |
| 37 | 0.714 | 0.422 | 1020 | 956 | 733 | 287 |
| 38 | 0.753 | 0.380 | 971 | 1005 | 751 | 220 |
| 39 | 0.768 | 0.349 | 956 | 1020 | 761 | 195 |
| 40 | 0.772 | 0.340 | 951 | 1025 | 764 | 187 |
| 41 | 0.775 | 0.338 | 948 | 1028 | 764 | 184 |
| 42 | 0.784 | 0.333 | 945 | 1031 | 769 | 176 |
| 43 | 0.784 | 0.324 | 934 | 1042 | 770 | 164 |
| 44 | 0.787 | 0.317 | 927 | 1049 | 773 | 154 |
| 45 | 0.788 | 0.314 | 924 | 1052 | 776 | 148 |
| 46 | 0.788 | 0.312 | 921 | 1055 | 776 | 145 |
| 47 | 0.790 | 0.311 | 921 | 1055 | 776 | 145 |
| 48 | 0.795 | 0.307 | 915 | 1061 | 777 | 138 |
| 49 | 0.795 | 0.303 | 911 | 1065 | 778 | 133 |
| 50 | 0.809 | 0.293 | 902 | 1074 | 784 | 118 |
| 51 | 0.826 | 0.272 | 892 | 1084 | 793 | 99 |
| 52 | 0.829 | 0.265 | 887 | 1089 | 795 | 92 |
| 53 | 0.831 | 0.260 | 881 | 1095 | 795 | 86 |
| 54 | 0.838 | 0.256 | 876 | 1100 | 795 | 81 |
| 55 | 0.840 | 0.256 | 876 | 1100 | 795 | 81 |
| 56 | 0.840 | 0.254 | 874 | 1102 | 795 | 79 |
| 57 | 0.844 | 0.251 | 872 | 1104 | 795 | 77 |
| 58 | 0.846 | 0.249 | 871 | 1105 | 796 | 75 |
| 59 | 0.849 | 0.245 | 868 | 1108 | 797 | 71 |
| 60 | 0.849 | 0.242 | 866 | 1110 | 797 | 69 |


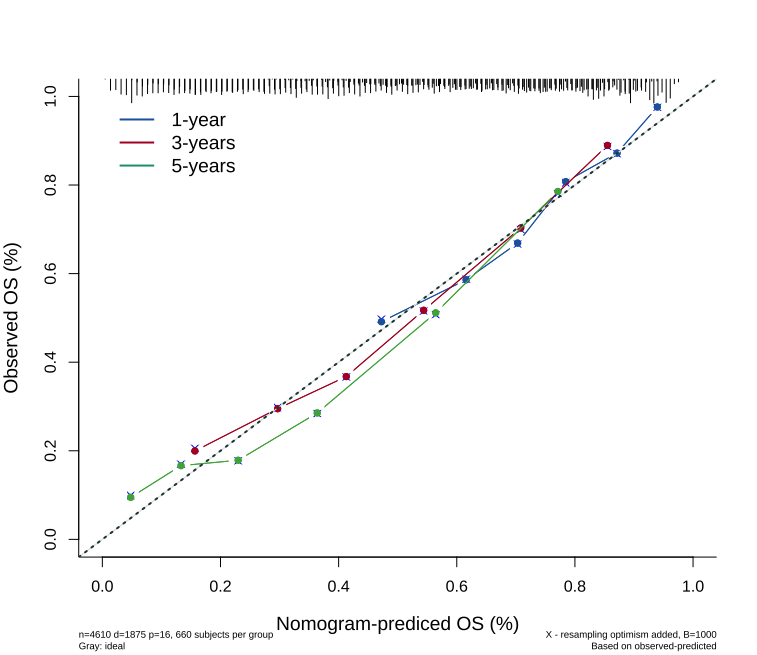


**Figure S 3 The 1-, 3-, and 5-year overall survival (OS) calibration curves of nomogram for non-small cell lung cancer patients.**


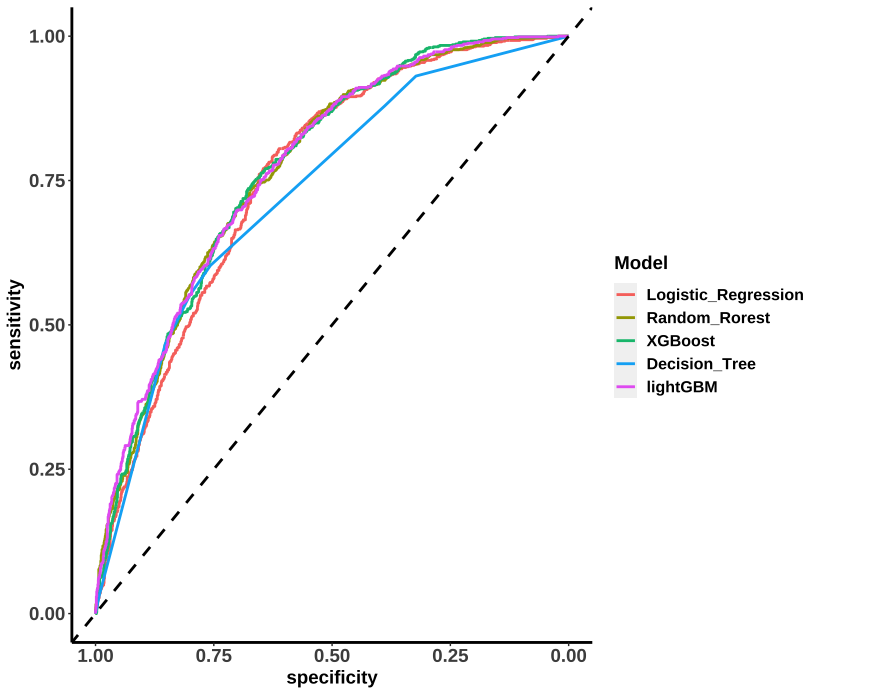


**Figure S 4 ROC curves of machine learning models.**
